# Amniocentesis and Risk of Fetal Loss in Dichorionic‐Diamniotic Twin Pregnancy: A Case‐Control Study

**DOI:** 10.1002/pd.6777

**Published:** 2025-03-18

**Authors:** Sofia Roero, Agata Ingala, Silvana Arduino, Carlotta Bossotti, Simona Bastonero, Francesca Maria Comoglio, Ilaria Dusini, Annasilvia Pertusio, Roberto Scali, Simona Sdei, Alberto Revelli, Andrea Sciarrone

**Affiliations:** ^1^ Gynaecology and Obstetrics 2U Sant’Anna Hospital Azienda Ospedaliero Universitaria Città della Salute e della Scienza di Torino University of Turin Turin Italy; ^2^ Obstetrics and Gynecological Ultrasound and Prenatal Diagnosis Center Sant’Anna Hospital Azienda Ospedaliero Universitaria Città della Salute e della Scienza di Torino Turin Italy; ^3^ Gynaecology and Obstetrics Department 3 Sant’Anna Hospital Azienda Ospedaliero Universitaria Città della Salute e della Scienza di Torino Turin Italy; ^4^ Gynaecology and Obstetrics 1U Sant’Anna Hospital Azienda Ospedaliero Universitaria Città della Salute e della Scienza di Torino University of Turin Turin Italy

## Abstract

**Objective:**

There is a paucity of data regarding the risk of fetal loss due to invasive prenatal diagnosis in twins. The aim of the present study is to assess the rate of amniocentesis‐related fetal loss in uncomplicated dichorionic‐diamniotic (DCDA) twin pregnancies.

**Methods:**

Retrospective observational case‐control study. DCDA twin pregnancies undergoing amniocentesis between January 2010 and December 2023 formed the case group. The control group comprised counterparts who did not undergo amniocentesis. The primary outcome of the study was procedure‐related fetal loss. Secondary outcomes were miscarriage rate, overall fetal loss and gestational age at birth.

**Results:**

Our dataset included 220 and 662 women in the case and control groups, respectively. No difference in the primary outcome was found: procedure‐related fetal loss of one fetus was 0.9% in the case group and 1.1% in the control group, and of both fetuses it was 0.5% in both groups (*p* = 0.982). No difference was found in secondary outcomes: the fetal loss rate of one fetus was 1.8% in the case group and 2.1% in the control group, while that of both fetuses it was 0.5% and 0.8% respectively (*p* = 0.853). Multivariate analysis confirmed the nonsignificant effect of amniocentesis on the risk of fetal loss.

**Conclusion:**

Amniocentesis does not seem to increase the risk of fetal loss in uncomplicated DCDA twin pregnancies above the baseline risk of loss among twin gestations.

**Summary**

- What's already known about this topic?
  ◦ Twin pregnancies have a higher background risk of fetal loss compared to singletons
  ◦ There are inconsistent data regarding the risk of fetal loss following invasive prenatal testing in twin pregnancies
- What does this study add?
  ◦ Amniocentesis does not seem to increase the rate of fetal loss above the intrinsic background risk of uncomplicated DCDA twin pregnancy
  ◦ Maternal age significantly affects the risk of overall fetal loss and miscarriage rate of DCDA twin pregnancy, whereas undergoing amniocentesis does not.

## 1 Introduction

The incidence of twin pregnancy has been rising in the last decades, mainly due to advanced maternal age and increased widespread availability of Medically Assisted Reproduction [1, 2]. Twin pregnancy is associated with an increased risk of maternal and perinatal mortality and morbidity [3, 4]. Dizygotic pregnancies are also more likely to be affected by chromosomal abnormalities compared with singletons: this is because the risk of chromosomal abnormalities applies to each fetus separately, thus making the global risk for the pregnancy twice that of singletons (as if the woman had two pregnancies in one). On the other hand, around 70% of monozygotic pregnancies result in monochorionic twins, who are more prone to structural malformations [5, 6]. As a consequence, prenatal diagnostic tools are essential in the context of twin pregnancy.

Noninvasive screening options include cell‐free fetal DNA testing (cffDNA) and the combined test (CT) based on a combination of maternal age, nuchal translucency and maternal serum markers. They have the advantage of non‐invasiveness, but their sensitivity and specificity appear to be slightly reduced in multiple pregnancies [7, 8, 9]. As regards cfDNA, in dizygotic twin pregnancy, each twin's DNA is distinct from that of their co‐twin, and each twin may contribute different amounts of cffDNA to the maternal circulation; there is a theorical risk that an euploid twin could contribute more cffDNA to the maternal circulation than the aneuploid co‐twin, leading to a false negative result [8]. Despite this, recent data suggest that the detection accuracy of trisomy 21 in twins is similar to that observed in singleton pregnancy [9, 10].

Invasive prenatal testing, either by chorionic villous sampling (CVS) or amniocentesis, remains the gold standard for the diagnosis of fetal aneuploidy or chromosomal abnormalities in twin pregnancy. However, procedure‐related complications—mainly the risk of fetal loss—represent the main concern about invasive testing. The rate of such complications is quite difficult to calculate, because the background risk of fetal loss is already higher in twin pregnancy compared to singletons. A recent meta‐analysis found that the rate of fetal loss before 24 weeks of gestation, or within 4 weeks after the procedure, did not differ from the background risk of twin pregnancies not undergoing invasive testing [11].

The heterogeneity of the currently available studies is quite significant, especially considering the definition of fetal loss and the wide range of indications for invasive prenatal testing. Indeed, it cannot be ignored that the risk of fetal loss is heavily influenced by the indication leading to the procedure as well as by the type of diagnostic procedure, the chorionicity, maternal characteristics and the mode of conception. Most available data come from studies involving both uncomplicated and complicated twin pregnancies without a clear separation on the basis of chorionicity, which greatly affects pregnancy outcome. Thus, there is a paucity of data regarding invasive prenatal testing in uncomplicated twin pregnancies, which would allow to exclude pregnancy loss related to chorionicity and common conditions such as growth restriction and malformations.

The aim of the present study is to assess the rate of amniocentesis‐related fetal loss in uncomplicated dichorionic‐diamniotic (DCDA) twin pregnancy, with the intention of gathering reliable data which could help patient counseling. Secondary outcomes include overall fetal loss, miscarriage rate and gestational age at birth.

## 2 Materials and Methods

### 2.1 Study Design

This retrospective case‐control study was carried out at Sant’Anna Women's Health Hospital in Turin (Italy) between January 2010 and December 2023. Because of anonymous data collection, the study was exempted from approval by the local institutional review board. The study fully adhered to the World Medical Association Declaration of Helsinki (as revised in 2013) and complied with the ethical standards of national and institutional committees on human experimentation. Written informed consent for use of personal information was obtained from every patient through a designated form that was signed at the time of the first visit. Furthermore, the present work fully adheres to the STROBE checklist for observational studies (Supporting Information S1).

### 2.2 Patients and Methods

Women with uncomplicated DCDA twin pregnancy undergoing amniocentesis between 16 and 20 weeks of gestational age (GA) in the study period formed the case group. The control group comprised women with uncomplicated DCDA twin pregnancy not undergoing IPT in the same study period.

Exclusion criteria were established in order to ensure selection of uncomplicated DCDA twin pregnancies and avoid any possible bias resulting from confounding maternal or fetal conditions. They were the following: previous CVS, selective fetal reduction or elective termination of pregnancy, increased nuchal translucency, ultrasound (US)‐diagnosed malformations, early intrauterine growth restriction (IUGR), chromosomal abnormalities, prenatal infection, history of recurrent miscarriages (3 or more previous miscarriages), and history of preterm birth. After application of the exclusion criteria, indications for invasive prenatal testing included advanced maternal age, maternal anxiety and maternal hemoglobinopathy and positive noninvasive prenatal screening.

Gestational age was calculated from the last menstrual period and confirmed by a first trimester ultrasound assessment, during which chorionicity was also established applying standard US criteria (i.e., presence of lambda sign) [12, 13]. At 19–21 weeks, a US scan for detailed anatomy assessment was performed. All patients with uncomplicated DCDA twin pregnancies underwent serial growth‐assessing US examinations every 4 weeks starting from week 20 as per the indications of national and international guidelines [12, 13, 14, 15]; if any anomaly was observed, US examinations were performed more frequently. As per our protocol, uncomplicated DCDA twin pregnancies were offered elective birth between 37 + 0 and 37 + 6 weeks of gestational age; the mode of delivery was evaluated for each patient considering fetal and obstetric indications.

AC was performed at our Center by expert operators (each one performing more than 100 procedures per year) between 16 and 20 weeks of gestational age.

The procedure was carried out with the woman lying supine on the bed; the abdomen was exposed. An amniocentesis instrument set was then opened and aseptic gloves were worn by the obstetrician. After disinfecting the skin, the abdomen was draped with sterile towels. The free‐hand ultrasound‐guided technique was adopted. A 20‐gauge needle was used and the first 2 mL of amniotic fluid aspirated was discarded. Each amniotic sac was tapped through a separate entry, and approximately 20 mL of amniotic fluid was drawn. All amniocentesis were performed via the transamniotic route. In around 7% ofcases (16/220), 5 mL of indigo carmine was instilled into the sac of the first twin that was sampled to confirm that separate sacs were sampled. After the procedure, fetal heart rates were assessed sonographically. Patients were asked to rest for 30 min to observe whether abdominal pain, severe uterine cramping, vaginal bleeding, or fluid leakage occurred.

### 2.3 Data Collection and Statistics

The following variables were recorded and analyzed: (a) women's characteristics: age, ethnicity, pre‐pregnancy body mass index (BMI); (b) primary outcome of the study: procedure‐related fetal loss (loss of one or both fetuses < 4 weeks of the procedure; in the control group, 4 weeks after 16 weeks); (c) secondary outcomes: miscarriage rate (< 24 weeks), overall fetal loss and gestational age at birth. With regard to gestational age at birth, newborn babies were divided into five groups: term delivery(> 37 weeks), late preterm delivery(between 34 and 37 weeks), moderate preterm delivery (between 28 and 34 weeks), and extremely preterm delivery (between 24 and 28 weeks).

The studied groups were compared using the two‐tailed Student's t‐test for parametric continuous variables, and the Mann‐Whitney *U* test for non‐parametric variables. Categorical data were compared using the *χ* ^2^ or Fisher's exact tests. We also performed multivariate logistic regression analysis in order to adjust for differences in maternal characteristics (i.e., maternal age, ethnicity and pre‐pregnancy BMI). Statistical analysis was performed using SPSS Statistical analysis software by SPSS Statistics Inc. IBM Corp (Released 2021. IBM SPSS Version 28.0.1.1). *p*‐values < 0.05 were considered statistically significant.

## 3 Results

**FIGURE 1:**
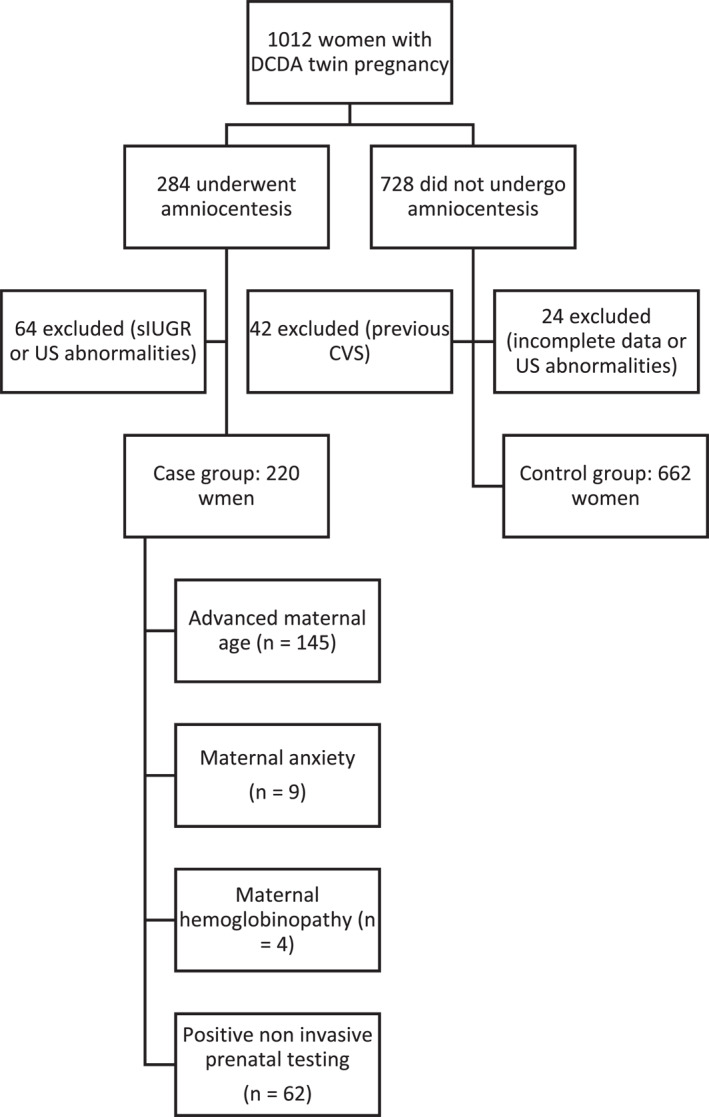
Flowchart of patients included in the study.

Among the 1012 women with DCDA twin pregnancy followed up at the Twin Pregnancy Care Unit of Sant’Anna Hospital during the study period, 284 underwent amniocentesis. 64 of them were excluded from the analysis because of IUGR or US abnormalities (increased nuchal translucency, malformations), and the case group was finally composed of 220 women. Among the remaining 728 women with uncomplicated DCDA twin pregnancy who did not undergo amniocentesis, 42 were excluded because they previously underwent CVS; the other 24 women were not included in the analysis because of incomplete data availability or ultrasound abnormalities; therefore, the control group included 662 women (Figure 1).

Table 1 shows maternal characteristics of the case and control groups. Women who underwent amniocentesis were significantly older (37.3 vs. 33.8 years; *p* < 0.001) and more often Caucasian (94.1% vs. 85.5%; *p* < 0.001) than those who did not. No significant difference in pre‐pregnancy maternal BMI was found. Around two thirds of women in our sample underwent amniocentesis because of maternal age, while in 28.2% of cases the indication was positive prenatal testing; maternal anxiety and maternal hemoglobinopathy accounted for a small fraction of indications.

**TABLE 1:** Maternal characteristics of the 882 pregnant women included in the study.

	Amniocentesis (*n* = 220)	No Amniocentesis (*n* = 662)	*p*‐value
Maternal age [years]	37.3 ± 4.1	33.8 ± 5.6	< 0.001
Maternal BMI^a^ [kg/m^2^]	23.3 ± 4.2	23.1 ± 4.3	0.543
Caucasian ethnicity	207 (94.1%)	564 (85.2%)	< 0.001
Advanced maternal age	145 (65.9%)	—	—
Maternal anxiety	9 (4.1%)	—	—
Maternal hemoglobinopathy	4 (1.8%)	—	—
Positive non invasive testing	62 (28.2%)	—	—

*Note:* Data are shown as mean ± SD or as number and percentage (in parenthesis).

^a^ Body Mass Index.

Table 2 shows a comparison of the primary outcome between the two groups. The rate of procedure‐related fetal loss was comparable between the case and the control groups: around 1% of women in each group lost one fetus, whereas 0.5% lost both (*p* = 0.982).

**TABLE 2:** Comparison of the primary outcomes between the case and control groups.

	Amniocentesis (*n* = 220)	No amniocentesis (*n* = 662)	*p*‐value
AC‐related fetal loss (1 fetus)	2 (0.9%)	7 (1.1%)	0.982
AC‐related fetal loss (both fetuses)	1 (0.5%)	3 (0.5%)	

*Note:* Data are shown as number and percentage (in parenthesis).

Table 3 shows the comparison of secondary outcomes between the two groups. Overall, the rates of fetal loss were quite low and did not differ significantly between women who underwent amniocentesis and those who did not. Around 2% of all considered women lost one fetus throughout pregnancy, whilst less than 1% lost both fetuses, without a significant difference between the two groups (*p* = 0.853). In the amniocentesis group, there was a smaller miscarriage rate than in the control group, both considering the miscarriage of one fetus (0.5% vs. 1.4%) and that of both fetuses (0.5% vs. 0.8%), but the difference was not significant (*p* = 0.427).

**TABLE 3:** Comparison of secondary outcomes between the case and control groups.

	Amniocentesis (*n* = 220)	No amniocentesis (*n* = 662)	*p*‐value
Overall fetal loss (1 fetus)	4 (1.8%)	14 (2.1%)	0.853
Overall fetal loss (both fetuses)	1 (0.5%)	5 (0.8%)	
Miscarriage (1 fetus)	1 (0.5%)	9 (1.4%)	0.427
Miscarriage (both fetuses)	1 (0.5%)	5 (0.8%)	

*Note:* Data are shown as number and percentage (in parenthesis).

Table 4 shows the comparison of gestational age at delivery between the two groups. Approximately 80% of twins were born after 34 weeks of gestational age; around 3% were delivered moderately preterm and 15% were extremely preterm in both groups. No significant difference was detected.

**TABLE 4:** Comparison of gestational age at birth between the case and control groups.

	Amniocentesis (*n* = 220)	No amniocentesis (*n* = 662)	
Delivery 24–27 weeks	37 (16.8%)	93 (14.0%)	0.349
Delivery 28–34 weeks	6 (2.7%)	15 (2.3%)	
Delivery 34–37 weeks	90 (40.9%)	322 (48.6%)	
Delivery > 37 weeks	86 (39.1%)	227 (34.3%)	
Double fetal loss	1 (0.5%)	5 (0.8%)	

*Note:* Data are shown as numbers and percentages (in parenthesis).

Multivariate analysis was performed in order to correct for possible bias deriving from differences in the population characteristics, such as maternal age and ethnicity. Results of the multivariate analysis are shown in Table 5. Maternal age was found to have a significant influence on both overall fetal loss and miscarriage rate (*p* = 0.024, OR 1.3, and 0.018, OR 1.4, respectively). No significant effect of amniocentesis on the three outcomes was observed.

**TABLE 5:** Multivariate analysis of the effects of maternal characteristics and AC procedure on the three main outcomes.

	OR (95% CI)	*p*‐value
Overall fetal loss		
Maternal age	1.3 (1.2–1.8)	0.024
BMI^a^	1.1 (0.9–1.2)	0.183
Ethnicity	1.4 (0.4–5.2)	0.573
Amniocentesis	0.9 (0.3–2.7)	0.557
Miscarriage rate		
Maternal age	1.4 (1.1–1.6)	0.018
BMI^a^	1.1 (0.9–1.2)	0.108
Ethnicity	1.6 (0.3–7.8)	0.389
Amniocentesis	0.3 (0.04–2.4)	0.194
Procedure‐related fetal loss		
Maternal age	1.1 (0.9–1.2)	0.281
BMI^a^	1.1 (0.9–1.3)	0.266
Ethnicity	1.9 (0.4–8.1)	0.564
Amniocentesis	1.0 (0.1–10.2)	0.432

^a^ Body Mass Index.

## 4 Discussion

Despite the relevant technological innovations in the context of noninvasive screening options, invasive prenatal testing remains a fundamental tool in the diagnosis of chromosomal abnormalities. This is even more true when considering twin pregnancy, as it is the only approach which allows for reliable and individual assessment of each twin. The accuracy of cff‐DNA testing in twin pregnancy, in fact, was recently reported to be quite high in detecting trisomy 21, but is still under evaluation for the other chromosomal abnormalities [10, 16]. For such reasons, invasive testing (amniocentesis and CVS) is still the gold standard in the diagnosis of genetic abnormalities in twin pregnancy and cff‐DNA testing is not recommended in case of US‐abnormality [8, 11]. In this context, the ever‐growing diagnostic capacity provided by US, together with the constantly increasing desire to receive reliable information regarding fetus' health, has led several women with a twin pregnancy to consider invasive prenatal testing. As a matter of fact, solid data (especially about safety) are needed to guarantee a correct counseling for these women, who face the decision whether or not to undergo IPT.

Several published studies about amniocentesis lack a control group composed of twin pregnancies not undergoing any invasive procedure [7, 17, 18, 19, 20, 21, 22, 23, 24]; they report the overall fetal loss rate and the amniocentesis‐related fetal loss rate without being able to compare them with a control group. As twin pregnancy implies an intrinsic increased risk of fetal loss compared to singleton pregnancies, studies lacking a control group provide very limited information about the possible additional risk deriving from amniocentesis [17, 25, 26].

This limitation has been partially solved by some case‐control studies, which however report rather inconsistent results. In fact, some studies reported an increased risk of fetal loss in twin pregnancies undergoing amniocentesis [27, 28, 29], while others did not find any significant increase above the background risk of twin pregnancy [30, 31, 32, 33, 34]. One possible explanation of such inconsistency might be the relevant heterogeneity of the studies: specifically, they differ for the definition of procedure‐related fetal loss, for exclusion criteria, and for the distribution of conditions potentially affecting fetal loss (malformations, history of miscarriage, positive noninvasive prenatal tests, etc.). Furthermore, in some of these studies [29, 34], the fetal loss rate in the amniocentesis group was underestimated due to the elective termination of pregnancy after the diagnosis of fetal aneuploidy.

Even the two systematic reviews that have been published are somehow inconsistent. Agarwal and Alfirevic [18] reported a fetal loss rate of 3.5% before 24 weeks for women undergoing amniocentesis, with an odd ratio of 1.8 compared to those not undergoing invasive testing. More recently, Di Mascio et al. [11] reported no significant increase in fetal loss rate before 24 weeks and within 4 weeks after AC, even if the overall fetal loss was slightly higher than the background risk of twin pregnancies, with an odd ratio of 1.46.

The case‐control study that we report here suggests that amniocentesis does not increase the risk of fetal loss above the intrinsic background risk of uncomplicated DCDA twin pregnancy. The rates we reported for miscarriage and procedure‐related fetal loss were 1% or less, approximately half of those reported in the recent metanalysis [11], probably due to our exclusion of pregnancies with abnormal US findings. However, the overall fetal loss that we observed was 2.1% and 1.8% in the amniocentesis and control groups, respectively, comparable to what was reported in the metanalysis (2.4% in both groups) [11]. In our multivariate analysis, a significant effect of maternal age on the risk of overall fetal loss and miscarriage rate was found, in agreement to what was reported by some studies [30] but not by others [7, 19]; differently, amniocentesis did not affect the fetal loss rates.

In our opinion, the only way to correctly calculate the net effect of amniocentesis on the risk of fetal loss is performing a priori an accurate selection of the cases that should be included in the analysis. In the present study, all pregnancies with abnormal US findings ranging from IUGR to increased nuchal translucency, suspected malformations, or maternal conditions potentially influencing the fetal loss rate were excluded. Indeed, Cai et al. reported an approximately four‐fold increase in the fetal loss rate in pregnancies with abnormal US findings [19]. Also, maternal and pregnancy characteristics such as ethnicity, increased nuchal translucency, and chorionicity have recently been shown to significantly influence the risk of fetal loss in twin pregnancies undergoing CVS [35], and it seems likely that this would apply to amniocentesis as well.

Despite all limitations linked to its retrospective nature, the present study included a relatively large cohort of patients homogeneously managed in a single Center; the application of strict exclusion criteria allowed to gather data on the effect of amniocentesis without any bias.

In conclusion, our data suggest that the net influence of amniocentesis on fetal loss in DCDA twin pregnancies seems to be relatively low. Overall, amniocentesis appears to be a relatively safe procedure and represents a feasible option for those women who want to have certain data regarding their offspring, with a low associated risk.

## Ethics Statement

The authors have nothing to report.

## Consent

Consent was obtained in written form from all patients for use of personal data. Regarding the gathering of information about neonatal outcomes, informed consent was obtained in written form from both patients.

## Conflicts of Interest

The authors declare no conflicts of interest.

## Supporting information

Supporting Information S1

## Data Availability

The data that support the findings of this study are available on request from the corresponding author. The data are not publicly available due to privacy or ethical restrictions.
