# Supplementary material for: Amniocentesis and Risk of Fetal Loss in Dichorionic‐Diamniotic Twin Pregnancy: A Case‐Control Study
Source: Prenat Diagn. 2025 Mar 18;45(6):713–9. doi: 10.1002/pd.6777 (PMC12137027; doi:10.1002/pd.6777)
Supplement: Supplementary file 1 — Supporting Information S1 [file PD-45-713-s001.doc]

STROBE Statement—Checklist of items that should be included in reports of ***case-control studies***

|  | Item No | Recommendation |
| --- | --- | --- |
| **Title and abstract** | 1 | (*a*) Indicate the study’s design with a commonly used term in the title or the abstract: *retrospective observational case-control study as indicated in the Title (page 1) and in the Abstract section (page 4 lines 85-103)* |
|  |  | (*b*) Provide in the abstract an informative and balanced summary of what was done and what was found  *Abstract is provided on page 4 lines 89-94.* |
| Introduction | | |
| Background/rationale | 2 | Explain the scientific background and rationale for the investigation being reported  *Page 5, lines 134-143* |
| Objectives | 3 | State specific objectives, including any prespecified hypotheses  *Page 5, lines 144-147* |
| Methods | | |
| Study design | 4 | Present key elements of study design early in the paper  *Page 5, line 153* |
| Setting | 5 | Describe the setting, locations, and relevant dates, including periods of recruitment, exposure, follow-up, and data collection  *Page 5-6, lines 153-154 (location and dates); lines 161-195 (setting, patient follow-up, protocols); lines 197-205 (data collection)* |
| Participants | 6 | (*a*) Give the eligibility criteria, and the sources and methods of case ascertainment and control selection. Give the rationale for the choice of cases and controls  *Page 6, lines 162-174* |
|  |  | (*b*) For matched studies, give matching criteria and the number of controls per case |
| Variables | 7 | Clearly define all outcomes, exposures, predictors, potential confounders, and effect modifiers. Give diagnostic criteria, if applicable  *Page 6-7, lines 198-205* |
| Data sources/ measurement | 8* | For each variable of interest, give sources of data and details of methods of assessment (measurement). Describe comparability of assessment methods if there is more than one group.  *Page 5, line 153-154; lines 174-195* |
| Bias | 9 | Describe any efforts to address potential sources of bias  *Page 6, lines 166-172* |
| Study size | 10 | Explain how the study size was arrived at  *Page 7, lines 215-222 + flowchart (table section, page 1)* |
| Quantitative variables | 11 | Explain how quantitative variables were handled in the analyses. If applicable, describe which groupings were chosen and why  *Page 7, lines 206-212* |
| Statistical methods | 12 | (*a*) Describe all statistical methods, including those used to control for confounding  *Page 7, lines 206-212* |
|  |  | (*b*) Describe any methods used to examine subgroups and interactions  *Not applicable.* |
|  |  | (*c*) Explain how missing data were addressed  *Not applicable.* |
|  |  | (*d*) If applicable, explain how matching of cases and controls was addressed  *Not applicable.* |
|  |  | (*e*) Describe any sensitivity analyses  *Not applicable.* |
| Results | | |
| Participants | 13* | (a) Report numbers of individuals at each stage of study—eg numbers potentially eligible, examined for eligibility, confirmed eligible, included in the study, completing follow-up, and analysed  *Page 7, lines 215-222* |
|  |  | (b) Give reasons for non-participation at each stage  *Page 7, lines 215-222* |
|  |  | (c) Consider use of a flow diagram  *A flowchart is provided (table section, page 1)* |
| Descriptive data | 14* | (a) Give characteristics of study participants (eg demographic, clinical, social) and information on exposures and potential confounders  *Page 7, lines 226-231* |
|  |  | (b) Indicate number of participants with missing data for each variable of interest  *Not applicable. Patients with missing data were excluded from the analysis.* |
| Outcome data | 15* | Report numbers in each exposure category, or summary measures of exposure  *Page 7, lines 215-222* |
| Main results | 16 | (*a*) Give unadjusted estimates and, if applicable, confounder-adjusted estimates and their precision (eg, 95% confidence interval). Make clear which confounders were adjusted for and why they were included  *Page 7, lines 226-264 (Tables 1-4)* |
|  |  | (*b*) Report category boundaries when continuous variables were categorized  *Tables 1-4.* |
|  |  | (*c*) If relevant, consider translating estimates of relative risk into absolute risk for a meaningful time period  *Not applicable.* |

| Other analyses | 17 | Report other analyses done—eg analyses of subgroups and interactions, and sensitivity analyses  *Reported in Table 5* |
| --- | --- | --- |
| Discussion | | |
| Key results | 18 | Summarise key results with reference to study objectives  *Page 8, lines 316-325* |
| Limitations | 19 | Discuss limitations of the study, taking into account sources of potential bias or imprecision. Discuss both direction and magnitude of any potential bias  *Lines 335-337; lines 326-330* |
| Interpretation | 20 | Give a cautious overall interpretation of results considering objectives, limitations, multiplicity of analyses, results from similar studies, and other relevant evidence  *Lines 338-342* |
| Generalisability | 21 | Discuss the generalisability (external validity) of the study results  *Lines 338-342* |
| Other information | | |
| Funding | 22 | Give the source of funding and the role of the funders for the present study and, if applicable, for the original study on which the present article is based  *Page 2, lines 48-49.* |

*Give information separately for cases and controls.

**Note:** An Explanation and Elaboration article discusses each checklist item and gives methodological background and published examples of transparent reporting. The STROBE checklist is best used in conjunction with this article (freely available on the Web sites of PLoS Medicine at http://www.plosmedicine.org/, Annals of Internal Medicine at http://www.annals.org/, and Epidemiology at http://www.epidem.com/). Information on the STROBE Initiative is available at http://www.strobe-statement.org.
